# A Rare Case of Sleep Terror Disorder in an Adult With Chronic Alcohol Abuse: A Case Report and Literature Review

**DOI:** 10.7759/cureus.41675

**Published:** 2023-07-11

**Authors:** Dhananjay Chaudhari, Nnesochi Okoroafor, Huzaifa Nadeem, Mandar Shah, Mihika A Shah, Mitsu Patel, Chinwe C Okonkwo, Pugazhendi Inban, Taha Sajjad, Aadil Khan

**Affiliations:** 1 Department of Psychiatry, Ganesh Shankar Vidyarthi Memorial Medical College and Lala Lajpat Rai Hospital, Kanpur, IND; 2 Department of Surgery, Imo State University Teaching Hospital, Orlu, NGA; 3 Department of Psychiatry, Combined Military Hospital (CMH) Lahore Medical College, Lahore, PAK; 4 College of Medicine, Smt. NHL Municipal Medical College, Ahmedabad, IND; 5 Department of Family Medicine, Caribbean Medical University School of Medicine, Willemstad, CUW; 6 Department of General Medicine, Government Medical College, Omandurar, Chennai, IND; 7 Department of Medical Education, Mountain Vista Medical Center, Phoenix, USA; 8 Department of Internal Medicine, Lala Lajpat Rai Hospital, Kanpur, IND

**Keywords:** polysomnography, insomnia, cognitive-behavioral therapy, chronic alcohol abuse, sleep terror disorder

## Abstract

Sleep terror disorder and chronic alcohol abuse are severe conditions that can significantly impact an individual's quality of life. Sleep terror disorder is characterized by sudden and intense episodes of fear or terror, while chronic alcohol abuse can lead to physical and psychological problems that can negatively impact sleep quality. This patient had terminal insomnia with episodes of terror, screaming, and no memory of arousal. Treatment of sleep terror disorder in chronic alcohol abuse patients involves addressing any underlying medical or psychological issues, medication, and cognitive-behavioral therapy (CBT). CBT can help identify and dispute harmful thought patterns and teach coping mechanisms. We present a case of an adult male who had terminal insomnia with episodes of terror, screaming, and no memory of arousal.

## Introduction

Sleep terror disorder is characterized by intense, sudden episodes of frightening or terror during sleep, usually accompanied by screaming, sweating, and rapid heart rate. The episodes typically occur during the first few hours of sleep and can last anywhere from a few seconds to a few minutes [1]. Sleep terror disorder is rare, affecting 1-7% of adults [2]. Chronic alcohol abuse has been known to disrupt the normal sleep cycle and lead to sleep disorders, including sleep terror disorder. Chronic alcohol abuse can cause significant changes in sleep architecture, leading to decreased rapid eye movement (REM) sleep, disrupted sleep-wake cycles, and altered sleep patterns [3]. The exact mechanism by which alcohol abuse leads to sleep terror disorder is not yet understood. Still, it is associated with the effect of alcohol on the brain and its ability to disrupt normal sleep patterns. Individuals with chronic alcohol abuse are at a higher risk of developing sleep terror disorder than the general population. Sleep terror disorder prevalence in individuals with alcohol use disorder is estimated to be between 20% and 36%. Sleep terror disorder in chronic alcohol abuse patients can significantly impact their quality of life and may lead to increased anxiety, depression, and other psychological problems [4].

Effective treatment of sleep terror disorder in chronic alcohol abuse patients typically involves addressing the underlying alcohol use disorder. Treatment may involve a combination of behavioral therapies, such as cognitive-behavioral therapy, relaxation techniques, and pharmacological interventions, such as benzodiazepines or antidepressants [5]. However, it is essential to note that treating sleep terror disorder in chronic alcohol abuse patients can be challenging and may require a multidisciplinary approach involving a team of healthcare professionals, including psychiatrists, psychologists, and sleep specialists [6].

## Case presentation

A 50-year-old male presented with complaints of fearfulness, screaming, and nightmares in the middle of the night for the past two years. His past medical history revealed headaches, anxiousness, and sleeplessness following a road accident in the past seven years, for which he was taking medication. He started to use alcohol (180 ml/day) for the past two years. Further history revealed he had terminal insomnia with periods of inconsolable terror, screaming, and irrelevant muttering with no memory of the arousal episodes. He underwent abrupt awakening, consistently transpiring within the initial three to four hours of sleep. The prevailing sensation manifested as intense agony, accompanied by subsequent effects such as a pounding heart, breathlessness, increased pressure in both ears, perspiration, and tense muscles in his limbs. Moments later, an overwhelming sense of something dreadful transpired. Promptly, his thoughts turned to his wife, only to discover her already awake and attempting to console him, at which point all symptoms gradually subsided. His wife recounted being startled by his scream, though the patient himself seemed unaware of it. He noted that the frequency of these episodes correlated significantly with his emotional state, particularly heightened anxiety concerning his wife's well-being. Additionally, the patient attested that a specific nightmare consistently preceded his awakening. The remaining physical and systemic examinations were normal. No significant family history of sleep disorder was present.

**Figure 1:**
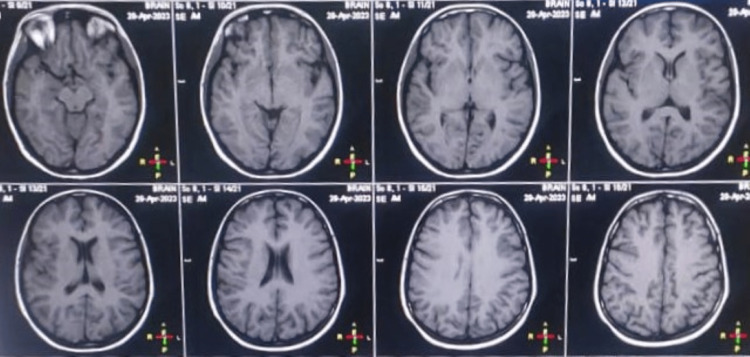
Normal brain MRI.

**Figure 2:**
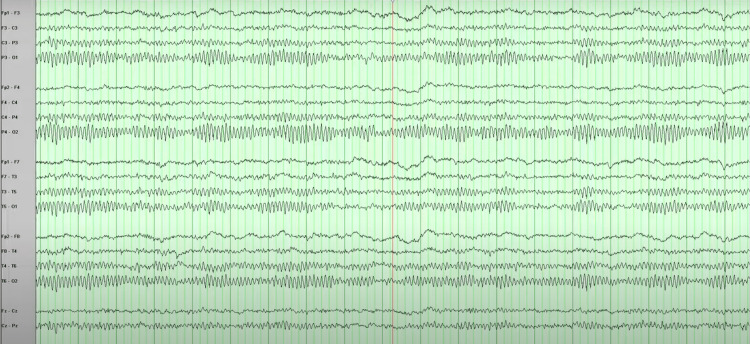
EEG with most of the waves of 8 Hz while awake demonstrating normal pattern.

On examination, he was hemodynamically stable. The systemic examination, including neurological examination, was within normal range. He underwent detailed evaluations, including thyroid function tests, hemogram, levels of vitamin B12, and toxic drug screening, which were within normal range. Viral markers and rheumatoid factors were also negative. Brain magnetic resonance imaging (MRI) and electroencephalography (EEG) revealed no abnormality (Figures 1, 2).

**Figure 3:**
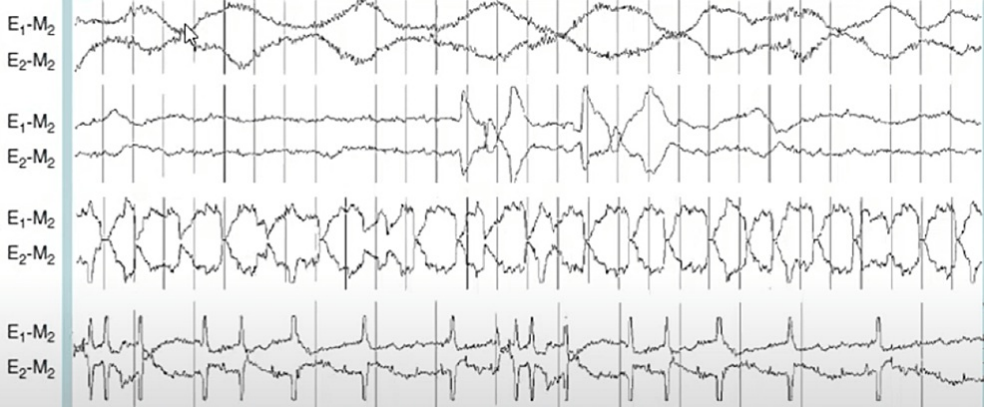
Polysomnography demonstrating sudden awakenings within the first 120 minutes of the N3 episode about two hours following the start of sleep during the second N3 sleep cycle.

A polysomnography revealed sudden awakenings within the first 120 minutes of non-rapid eye movement (NREM) sleep that was staged as part of a sleep stage 4 (now N3) episode about two hours following the start of sleep during the second N3 sleep cycle, leading to the diagnosis of night terrors (Figure 3). REM sleep was not associated with any anomalies that would suggest an epileptic-like condition. Owing to the above diagnosis, the patient was started on a therapeutic regimen of naltrexone at 50 mg per day and oral thiamine at 300 mg per day for five days for underlying chronic alcoholism. In addition, quetiapine 50 mg twice a day with melatonin 5 mg once a day was also added, and the patient was reassured and counseled for sleep hygiene and followed up after two weeks. On the next visit, the patient was satisfied with the treatment given and noticed fewer arousal episodes in the night with good sleep and a significant cut down in his alcohol intake.

## Discussion

Sleep terror disorder and chronic alcohol abuse are serious conditions that can significantly impact an individual's quality of life. When these two conditions co-occur, the situation can become even more challenging. Sleep terror disorder is a type of parasomnia that typically occurs during NREM sleep and is characterized by sudden and intense episodes of fear or terror. Chronic alcohol abuse, on the other hand, can lead to physical and psychological problems that can negatively impact sleep quality [7]. There are various ways in which chronic alcohol abuse can lead to the development of sleep terror disorder. Firstly, alcohol can disrupt normal sleep patterns and cause changes in brain chemistry that may contribute to developing sleep disorders. Secondly, alcohol can increase anxiety and stress levels, worsening sleep terror disorder symptoms. Thirdly, chronic alcohol abuse can lead to physical health problems such as liver disease and sleep apnea, which can also contribute to developing sleep disorders.

Insomnia with sleep terror disorder is a prevailing and enduring issue that occurs throughout various stages of alcohol dependence, impacting both the subjective and objective aspects of sleep. It is highly prevalent among individuals with alcohol dependence, with estimates ranging from 36% to 91% [8]. Insomnia is present across different stages of alcohol dependence and is associated with each of these stages. Patients experiencing active alcohol use and dependence often encounter insomnia, with rates as high as 74%. Insomnia symptoms include increased sleep latency, fragmented sleep, and reduced total sleep time. Among veterans with alcohol dependence, insomnia symptoms such as prolonged time to fall asleep, wakefulness after sleep onset, and impaired sleep quality are common, particularly among those with concurrent psychiatric disorders. Sleep complaints during the acute withdrawal phase can vary in prevalence. Studies suggest that the majority of inpatients with alcohol dependence (92%) experience sleep disturbances during acute withdrawal. Subjective sleep disruption is particularly prevalent among women (100%) and men (88.9%) undergoing inpatient alcohol detoxification [9]. During the recovery phase from alcohol use, insomnia continues to persist, affecting approximately 65% of individuals. Subjective complaints include increased time to fall asleep, heightened wakefulness after sleep onset, and reduced sleep efficiency. Objective measures reveal increased sleep latency, elevated stage 1 sleep, decreased total sleep time, and alterations in REM sleep. The relationship between relapse and insomnia is inconclusive. Over time, REM sleep abnormalities may normalize [10].

This study includes a 50-year-old male who visited the psychiatry outpatient department with a two-year history of fearfulness, screaming, and nightmares during the night. The patient had a medical history of anxiety, sleeplessness, and headaches after a road accident seven years ago, for which he took medication. However, he started consuming 180 ml of alcohol daily two years ago, exacerbating his symptoms. The patient exhibited episodes of terminal insomnia with inconsolable terror, screaming, and amnesia, lasting five to eight minutes, accompanied by symptoms of agitation, palpitations, sweating, and rapid breathing, while physical examinations were unremarkable and no significant family history of sleep disorders was reported.

Ambulatory polysomnography was conducted with the patient's consent to confirm the diagnosis, revealing sudden awakenings within the first 120 minutes of NREM sleep, staged as part of a sleep stage 4 (now N3) episode. The diagnosis was night terrors, and no anomalies during REM sleep suggested an epileptic-like condition. The patient has been prescribed quetiapine 50 mg twice a day with melatonin 5 mg once a day, and a therapeutic regimen for chronic alcoholism. Additionally, the patient was advised to follow sleep hygiene and was monitored for two weeks. He reported reduced alcohol intake and fewer arousal episodes, improving sleep quality. The patient expressed satisfaction with the treatment received.

The treatment of sleep terror disorder in chronic alcohol abuse patients can be challenging, as both conditions need to be addressed simultaneously. Treatment typically involves identifying and addressing any underlying medical or psychological issues contributing to the problem. In the case of a chronic alcohol abuse patient with sleep terror disorder, treatment may involve addressing the alcohol abuse through detoxification and rehabilitation programs and addressing any co-occurring mental health conditions such as anxiety or depression. Medications such as benzodiazepines or antidepressants may also be prescribed to help manage symptoms. Benzodiazepines are typically used to reduce anxiety and help patients relax, while antidepressants can help regulate mood and improve sleep quality. However, it is essential to note that benzodiazepines and antidepressants can be habit-forming and should only be used under the guidance of a medical professional. CBT can also help treat sleep terror disorder in chronic alcohol abuse patients. CBT can assist individuals in recognizing and disputing harmful thought patterns that could be playing a role in their symptoms., as well as teach them coping mechanisms and relaxation techniques to manage their symptoms [11].

## Conclusions

Although rare, individuals with chronic alcohol abuse are at a higher risk of developing sleep terror disorder. It can have a significant impact on quality of life and lead to increased anxiety, depression, and other psychological problems. We properly evaluated and managed the patient, who reacted well to the treatment and was closely monitored. The takeaway from this case is that sleep terror conditions and chronic alcohol consumption can coexist, thus history and symptoms must be studied to rule out sleep terror disorder.
